# Impact of rapid iodine contrast agent infusion on tracheal diameter and lung volume in CT pulmonary angiography measured with deep learning-based algorithm

**DOI:** 10.1007/s11604-024-01591-7

**Published:** 2024-05-11

**Authors:** Koichiro Yasaka, Osamu Abe

**Affiliations:** Department of Radiology, The University of Tokyo Hospital, 7-3-1 Hongo, Bunkyo-ku, Tokyo, 113-8655 Japan

**Keywords:** CT pulmonary angiography, Iodine contrast agent, Rapid infusion, Tracheal diameter, Lung volume

## Abstract

**Purpose:**

To compare computed tomography (CT) pulmonary angiography and unenhanced CT to determine the effect of rapid iodine contrast agent infusion on tracheal diameter and lung volume.

**Material and methods:**

This retrospective study included 101 patients who underwent CT pulmonary angiography and unenhanced CT, for which the time interval between them was within 365 days. CT pulmonary angiography was scanned 20 s after starting the contrast agent injection at the end-inspiratory level. Commercial software, which was developed based on deep learning technique, was used to segment the lung, and its volume was automatically evaluated. The tracheal diameter at the thoracic inlet level was also measured. Then, the ratios for the CT pulmonary angiography to unenhanced CT of the tracheal diameter (TD_PAU_) and both lung volumes (BLV_PAU_) were calculated.

**Results:**

Tracheal diameter and both lung volumes were significantly smaller in CT pulmonary angiography (17.2 ± 2.6 mm and 3668 ± 1068 ml, respectively) than those in unenhanced CT (17.7 ± 2.5 mm and 3887 ± 1086 ml, respectively) (*p* < 0.001 for both). A statistically significant correlation was found between TD_PAU_ and BLV_PAU_ with a correlation coefficient of 0.451 (95% confidence interval, 0.280–0.594) (*p* < 0.001). No factor showed a significant association with TD_PAU_. The type of contrast agent had a significant association for BLV_PAU_ (*p* = 0.042).

**Conclusions:**

Rapid infusion of iodine contrast agent reduced the tracheal diameter and both lung volumes in CT pulmonary angiography, which was scanned at end-inspiratory level, compared with those in unenhanced CT.

## Introduction

The iodine contrast agent is widely used in computed tomography (CT) examinations. However, some adverse reactions associated with iodine contrast agent infusion exist, such as hypersensitivity reaction [1], contrast-induced nephropathy [2] and metformin-induced lactic acidosis [3] among patients with renal function impairment. In addition to these adverse reactions, we coincidentally found another phenomenon related to iodine contrast agent rapid infusion, i.e., a decrease of tracheal diameter and both lung volume in the early phase [4], in addition to these already-known adverse reactions.

With the advancement in deep learning applications in the field of radiology [5–7], lung volumetry can now be performed more easily and accurately [8–10]. Lung volumetry has an important role in the clinical management of lung diseases. For example, relative annual bilateral lung volume loss was reportedly high in patients with interstitial lung disease with major adverse events at a 3-year follow-up compared with those without [11]. Additionally, CT lung volumetry is an effective method for appropriate size matching between donor and recipient in lung transplantation [12]. Moreover, CT lung volumetry is useful for some other conditions, including bronchiolitis obliterans detection in patients after lung transplantation [13], chronic obstructive pulmonary disease [14, 15], and coronavirus pneumonia [16]. Airway diameter is also known to play an important role in some diseases, especially asthma. A bronchodilator is used to manage this asthma, which is characterized by loss of homeostatic control of the airway smooth muscle [17]. Therefore, accurate measurements of lung volume and tracheal diameter are deemed to be necessary for some disease management as described above. A recent study, which included patients who underwent vascular dynamic CT examination, has revealed that a decrease of tracheal diameter and both lung volume can be seen in the arterial phase [4]. However, data regarding the impact of iodine contrast agent rapid infusion on tracheal diameter and lung volume in CT pulmonary angiography, for which the scan delay is shorter than the arterial phase and various patients with various backgrounds are included, are lacking.

This study aimed to investigate the impact of rapid iodine contrast agent infusion on tracheal diameter and lung volume in CT pulmonary angiography by comparing it with unenhanced CT.

## Materials and methods

Our Institutional Review Board, which waived the requirement for obtaining written informed consent from patients, approved this retrospective study.

### Patients

We searched picture archiving and communication systems for patients who met all the following inclusion criteria: (a) Those who underwent CT pulmonary angiography from January 2021 to December 2021, (b) Those for whom unenhanced CT image within 365 days from the CT pulmonary angiography is available, and (c) Those without acute lung or pleural lesions such as consolidation or pleural effusion at both the CT pulmonary angiography and unenhanced CT. Patients for whom segmentation errors occurred were excluded from the study (*n* = 1).

### CT imaging

Scanners from two vendors (Canon Medical Systems [Tochigi, Japan] and GE Healthcare [WI, US]) were used for CT examinations. Scan and reconstruction parameters were the following: tube current, automatic tube current modulation with standard deviation/noise index set at 13.0/11.36–11.4 for Canon-CT/GE-CT; tube voltage, 120 kVp except for CT pulmonary angiography with Revolution CT scanner from GE-CT in which dual-energy CT was performed and 70 keV monochromatic image was reconstructed; gantry rotation time, 0.5 s; helical pitch, 0.813/1.375/0.992 for Canon-CT/GE-CT (other than Revolution CT)/GE-CT (Revolution CT); field of view, 350 mm; slice thickness/interval, 5 mm/5 mm. A previous report revealed that CT attenuation in 70 keV monochromatic CT images corresponds to those in 120 kVp CT [18].

In our institution, breath hold instruction for the CT pulmonary angiography has been changed from end-inspiratory level to mid-inspiratory level in January 2022 to reduce a risk for transient interruption of contrast. Therefore, all the patients included in this study, which included those who underwent CT pulmonary angiography from January 2021 to December 2021, were instructed to hold their breath at an end-inspiratory level during scans in both the CT pulmonary angiography and unenhanced CT examinations in this study. For CT pulmonary angiography, an iodine contrast agent was injected from the right or left antecubital vein within 30 s using an automatic power injector for CT pulmonary angiography. Breath hold started after contrast injection. Immediately after the breath hold, CT scan was started. Patients were scanned 20 s after the initiating contrast injection. The molecule name of iodine contrast agents used in this study included iomeprol (Bracco), iopamidol (Bayer), iohexol (GE Healthcare), and ioversol (Guerbet). For unenhanced CT, CT scan was also started immediately after the breath hold.

### CT image evaluation

**Fig. 1:**
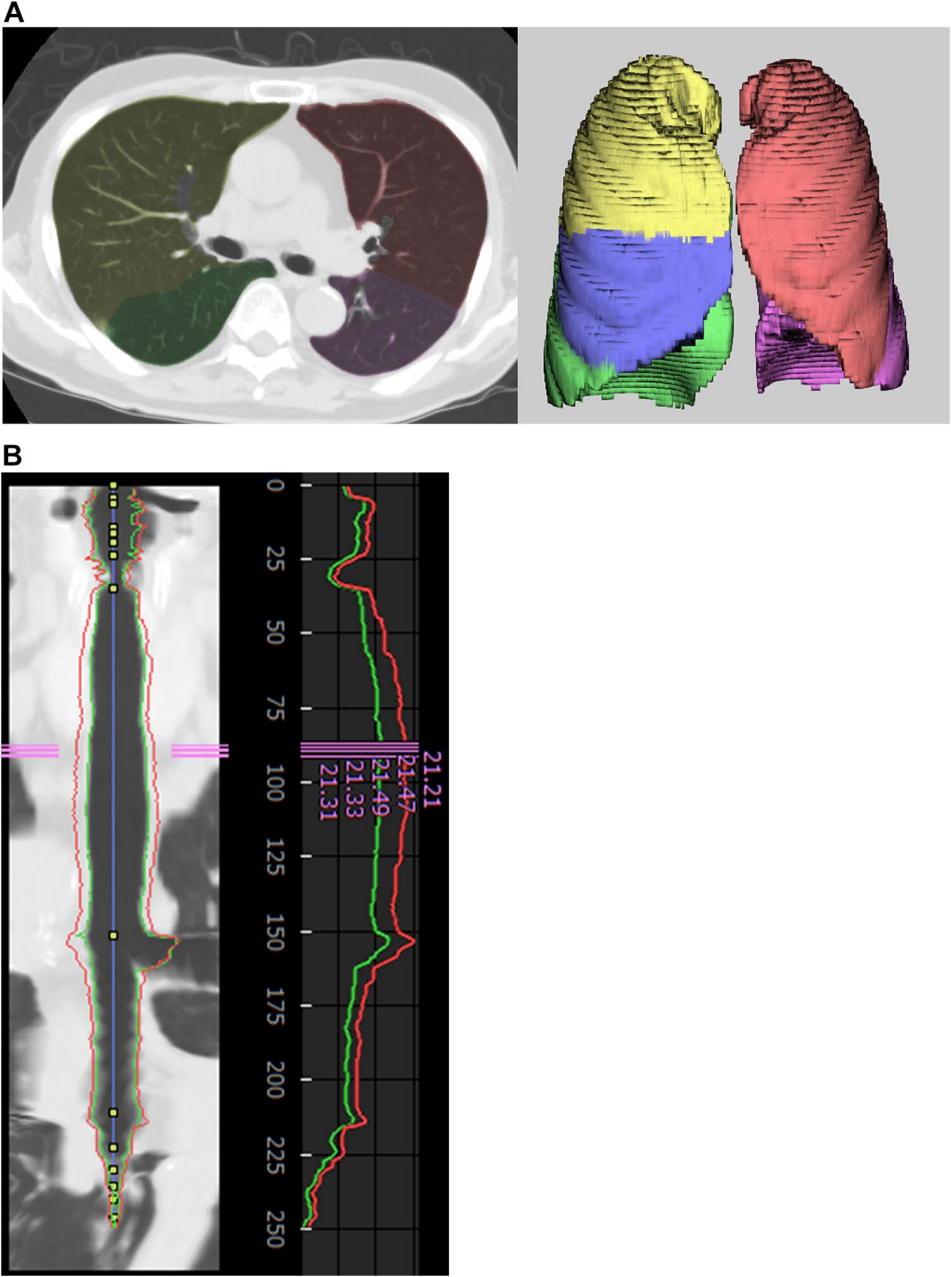
Evaluations of **A** lung volume and **B** tracheal diameter with commercial software. **A** The right upper, right middle, right lower, left upper, and left lower lobes were segmented and highlighted with yellow, blue, green, red, and pink, respectively. **B** The internal lumen and outside boundary of the airway are delineated with green and red color curved lines, respectively. The average internal lumen diameter is displayed with pink numerals

A radiologist with diagnostic imaging experience of 13 years evaluated the CT images with commercial software [Synapse Vincent, Fujifilm (Tokyo, Japan)], which was developed based on deep learning technique. First, each lobe was fully automatically segmented (Fig. 1A), and the volume of each lobe as well as those of the right, left, and both lungs were calculated. The radiologist checked whether segmentation was performed correctly and recorded these volumes. Then, airway segmentation was also performed with this software (Fig. 1B). The mean of the inner lumen diameter was displayed, and the radiologist recorded the tracheal inner lumen diameter at the thoracic inlet level.

The ratios for the CT pulmonary angiography to unenhanced CT of the tracheal diameter (TD_PAU_), both lung volume (BLV_PAU_), and each lobe volume were calculated.

### Statistical analysis

R version 4.1.2 (https://www.r-project.org/) was used for statistical analyses. Tracheal diameter and lung volume were compared between CT pulmonary angiography and unenhanced CT with paired *t* tests. Student’s *t* test compared the ratio for upper and middle lobes *vs.* lower lobes. Pearson’s correlation coefficient assessed the associations between TD_PAU_ *vs.* BLV_PAU_ and TD_PAU_ or BLV_PAU_ *vs.* age. Student’s *t *test or analysis of variance compared TD_PAU_ and BLV_PAU_ values between categories for factors with nominal variables. Post-hoc test with Bonferroni correction was performed for significant factors with analysis of variance. *P *values of < 0.05 were considered to indicate statistical significance.

## Results

### Patients

**Fig. 2:**
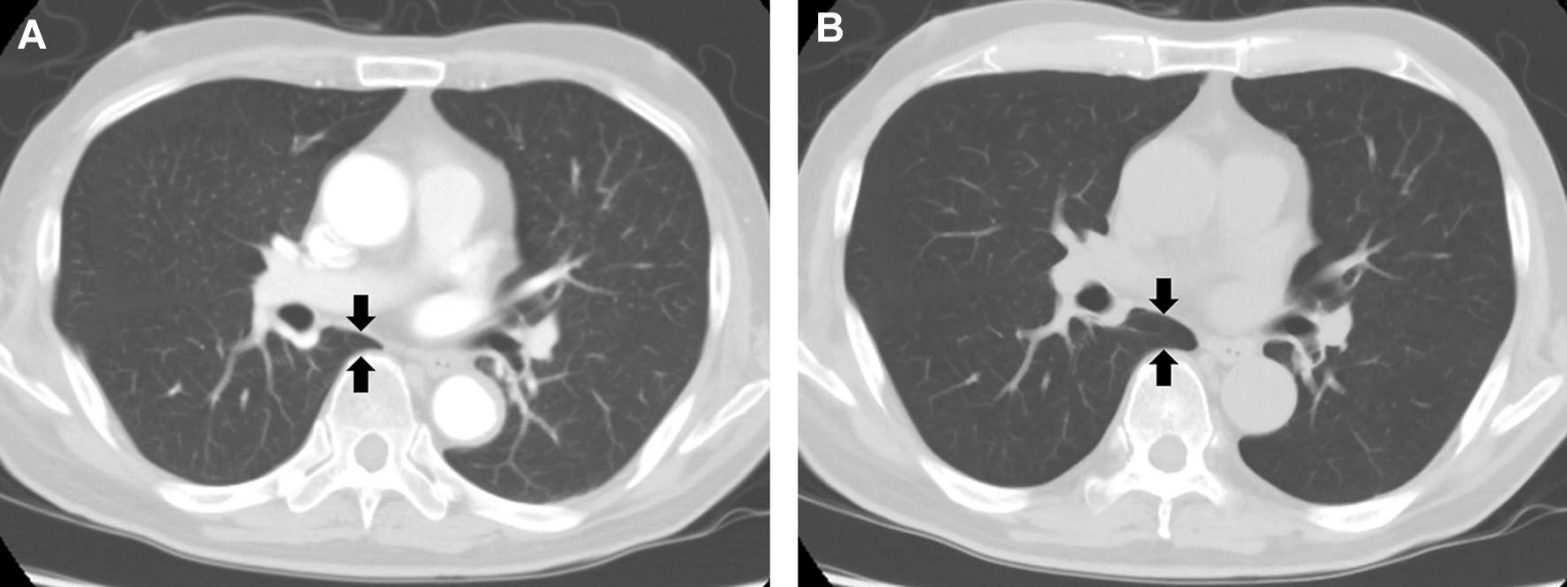
CT images of a 73-year-old male patient who was injected with iopamidol at 300 mgI/ml from the right antecubital vein. This patient underwent both the CT pulmonary angiography and unenhanced CT on the same day. Tracheal diameter/both lung volume was 19.0 mm/3943 ml in CT pulmonary angiography **A** and 21.0 mm/4807 ml in unenhanced CT. **B** Reduction of lung volume can be visually recognized with the reduction of anteroposterior diameter of the azygoesophageal recess (black arrows)

This study included 101 patients (mean age, 64.8 ± 16.0 years; 41 males). The clinical indication for the CT pulmonary angiography was the evaluations of pulmonary embolism for all patients. Patient background diseases were gynecologic malignancy (*n* = 17), urological malignancy (*n* = 9), gastrointestinal malignancy (*n* = 9), hepatobiliary malignancy (*n* = 5), interstitial pneumonitis (*n* = 5), lung carcinoma (*n* = 4), breast carcinoma (*n* = 4), hematological malignancy (*n* = 4), post operative state for total hip or knee arthroplasty (*n* = 3), no significant background disease (*n* = 21), and others (*n* = 20). Table 1 shows other patient background information. Figure 2 shows representative CT pulmonary angiography and unenhanced CT images.

**Table 1:** Patient background information

	Value
Number of patients	101
Age (average ± standard deviation) (years)	64.8 ± 16.0
Sex	
Male	41
Female	60
Time interval between CT pulmonary angiography and unenhanced CT (median with interquartile range) (days)	50 (15–127)
Pulmonary embolism	
Present	23
Absent	78
The side of contrast injection	
Right	71
Left	30
Contrast agent	
Iomeprol 350 mgI/ml	12
Iopamidol 300 mgI/ml	4
Iopamidol 370 mgI/ml	43
Iohexol 350 mgI/ml	37
Ioversol 320 mgI/ml	5

### CT imaging

The numbers of CT pulmonary angiography and unenhanced CT performed using Canon-CT/GE-CT (other than Revolution CT)/GE-CT (Revolution CT) were 46/17/38 and 49/2/50, respectively. Unenhanced CT was performed from July 2020 to September 2022 (53/6/42 patients for before/same day as/after the CT pulmonary angiography). The CT dose index volume for the CT pulmonary angiography and unenhanced CT was 8.09 ± 2.79 mGy and 9.27 ± 3.52 mGy, respectively. There was no statistically significant difference in tube current between CT pulmonary angiography and unenhanced CT (*p* = 0.637, Wilcoxon signed rank test). Although there was a statistically significant difference in helical pitch between CT pulmonary angiography and unenhanced CT (*p* = 0.002, Wilcoxon signed rank test), helical pitch did not have a significant impact on tracheal diameter or both lung volume in CT pulmonary angiography (r = 0.046 [*p* = 0.651] or r = 0.073 [*p* = 0.471], respectively) and unenhanced CT (r = 0.026 [*p* = 0.798] or r = 0.072 [*p* = 0.476], respectively) with Spearman’s rank correlation coefficient analysis.

### Effect of iodine contrast infusion on tracheal diameter and both lung volume

Tracheal diameter in CT pulmonary angiography was 17.2 ± 2.6 mm which was significantly smaller than those in unenhanced CT (17.7 ± 2.5 mm) (*p* < 0.001) (Table 2). The tracheal diameter in CT pulmonary angiography was decreased by 2.8% compared with unenhanced CT. More than a 10% decrease was seen in 4.0% of patients (Table 3).

**Table 2:** Tracheal diameter and both lung volumes in CT pulmonary angiography and reference unenhanced CT

	CT pulmonary angiography	Unenhanced CT	*P *value
Tracheal diameter (mm)	17.2 ± 2.6	17.7 ± 2.5	< 0.001^*^
Lung volume (ml)			
Both lung	3668 ± 1068	3887 ± 1086	< 0.001^*^
Right lung	2027 ± 569	2118 ± 599	0.004^*^
Right upper lobe	786 ± 258	815 ± 263	< 0.001^*^
Right middle lobe	59 ± 118	361 ± 120	0.749
Right lower lobe	881 ± 320	942 ± 336	0.005^*^
Left lung	1642 ± 521	1770 ± 514	< 0.001^*^
Left upper lobe	938 ± 305	988 ± 293	< 0.001^*^
Left lower lobe	704 ± 273	782 ± 280	< 0.001^*^

Mean ± standard deviation values are described

^*^Statistically significant

**Table 3:** Percentage of patients based on TD_PAU_ and BLV_PAU_ values

	TD_PAU_	BLV_PAU_
Average	0.972 ± 0.048	0.952 ± 0.146
< 0.9	4.0% (4/101)	35.6% (36/101)
< 0.8	0.0% (0/101)	10.9% (11/101)
> 1.1	1.0% (1/101)	13.9% (14/101)
> 1.2	0.0% (0/101)	5.0% (5/101)

BLV_PAU_, ratio of CT pulmonary angiography to unenhanced CT for both lung volume; TD_PAU_, ratio of CT pulmonary angiography to unenhanced CT for tracheal diameter

Both lung volumes in CT pulmonary angiography were 3668 ± 1068 ml which was significantly smaller than those in unenhanced CT (3887 ± 1086 ml) (*p* < 0.001) (Table 2). Both lung volumes in CT pulmonary angiography were decreased by 4.8% compared with unenhanced CT. More than 10% and 20% decrease were seen in 35.6% and 10.9% of patients, respectively (Table 3).

**Fig. 3:**
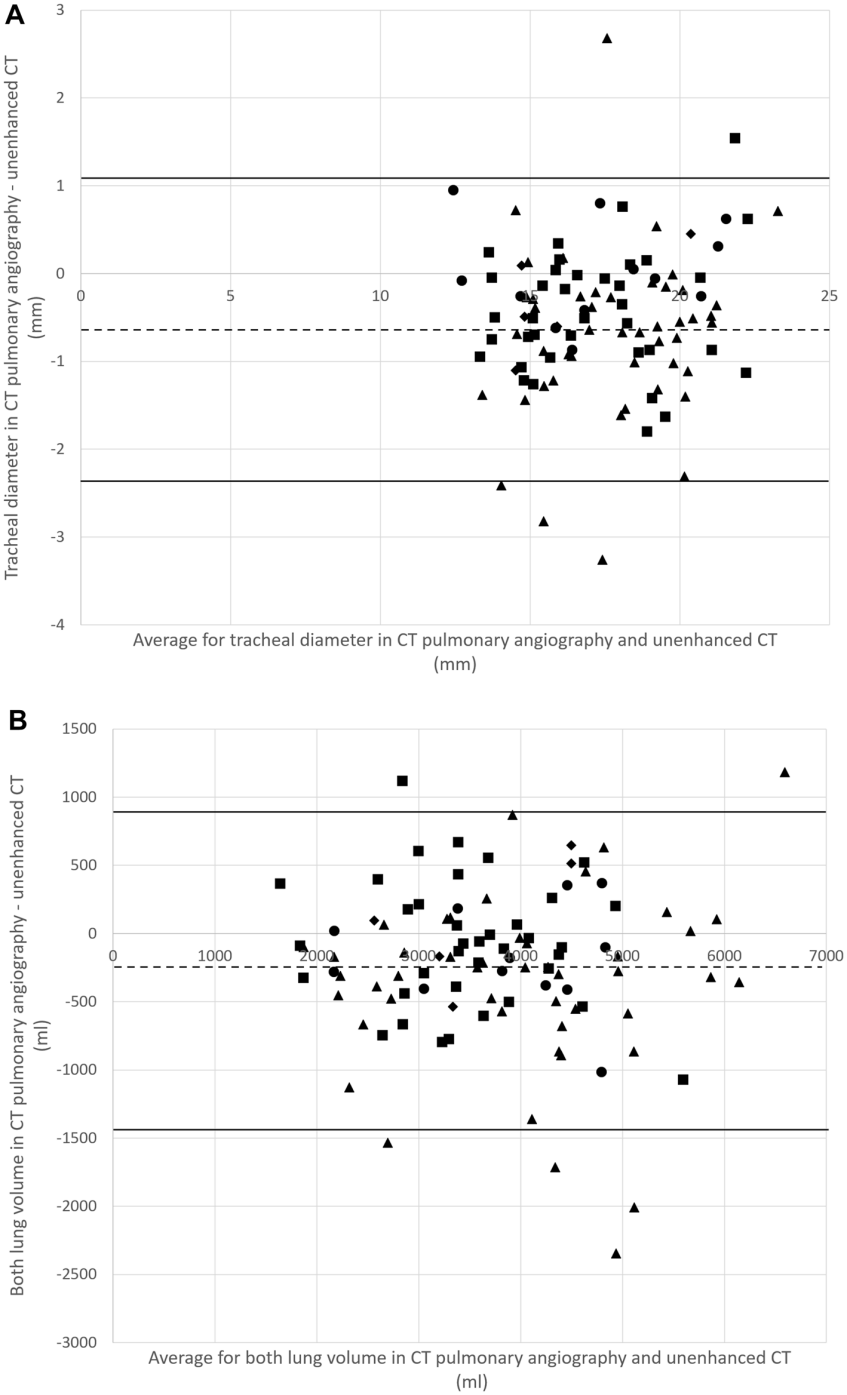
Bland Altman plots between CT pulmonary angiography and unenhanced CT for the **A** tracheal diameter and **B** both lung volume. Dotted and solid lines indicate mean difference and limit of agreement, respectively. Circle, triangle, square, and diamond markers indicate iomeprol, iopamidol, iohexol, and ioversol, respectively

Figure 3 shows Bland Altman plots between CT pulmonary angiography and unenhanced CT for the tracheal diameter and both lung volumes.

Ratios of CT pulmonary angiography to unenhanced CT for the right upper, right middle, right lower, left upper, and left lower lobe volumes were 0.968 ± 0.147, 1.006 ± 0.123, 0.963 ± 0.230, 0.945 ± 0.117, and 0.921 ± 0.221, respectively. The ratio for lower lobes (0.942 ± 0.226) was significantly smaller than those of upper or middle lobes (0.973 ± 0.116) (*p* = 0.046).

**Fig. 4:**
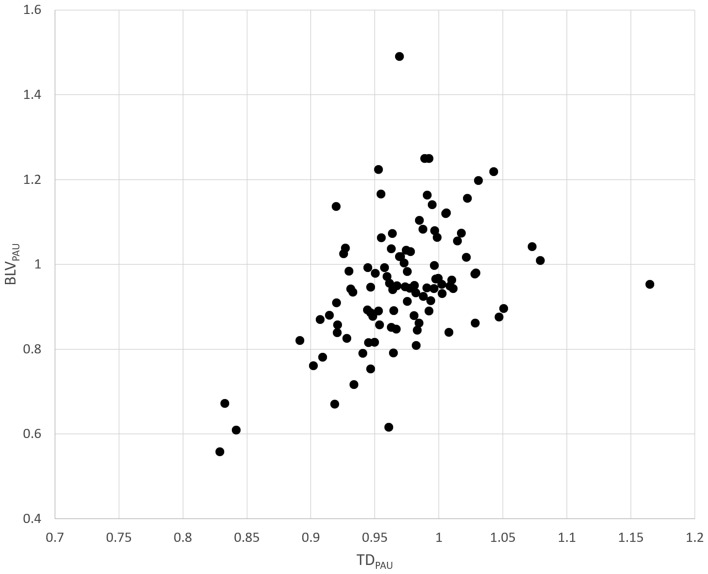
Relation between TD_PAU_ and BLV_PAU_. BLV_PAU_, the ratio of CT pulmonary angiography to unenhanced CT for both lung volumes; TD_PAU_, the ratio of CT pulmonary angiography to unenhanced CT for tracheal diameter

A statistically significant correlation was found between TD_PAU_ and BLV_PAU_ with a correlation coefficient of 0.451 (95% confidence interval, 0.280–0.594) (*p* < 0.001) (Fig. 4).

### Factors affecting the ratio of CT pulmonary angiography to unenhanced CT for the tracheal diameter and both lung volumes

**Fig. 5:**
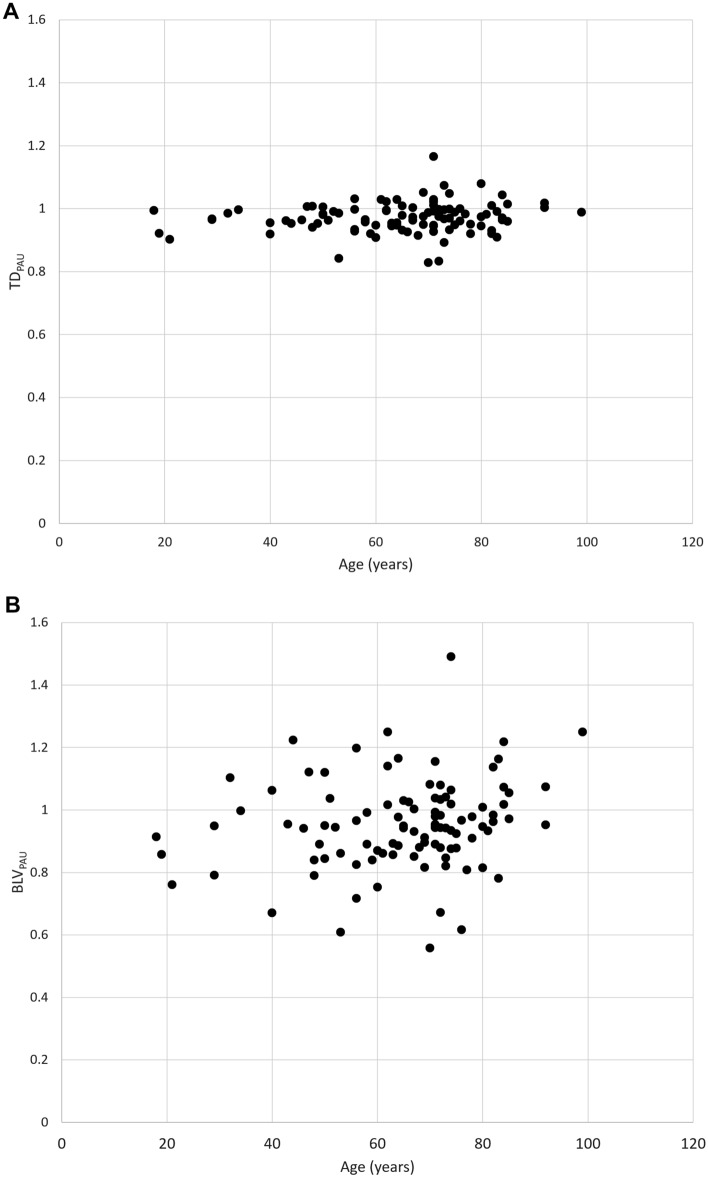
Relation between age *vs.* TD_PAU_ **A** and BLV_PAU_. **B** BLV_PAU_, the ratio of CT pulmonary angiography to unenhanced CT for both lung volumes; TD_PAU_, the ratio of CT pulmonary angiography to unenhanced CT for tracheal diameter

Table 4 shows the associations between each factor vs. TD_PAU_ and BLV_PAU_. Figure 5 shows the association between age vs. TD_PAU_ and BLV_PAU_. No factor had a significant association with TD_PAU_. The type of contrast agent had a significant association with BLV_PAU_ (*p* = 0.042). BLV_PAU_ (0.910) was smaller than other contrast agents (0.956–1.023) when iopamidol was used in CT pulmonary angiography, and a statistically significant difference was observed between iopamidol and iohexol (*p* = 0.038).

**Table 4:** Associations between each factor *vs.* TD_PAU_ and BLV_PAU_

	TD_PAU_		BLV_PAU_	
	Value	*P *value	Value	*P *value
Age	0.146 (−0.051–0.332)	0.146	0.189 (−0.006–0.371)	0.058
Sex		0.710		0.652
Male	0.973 ± 0.044		0.960 ± 0.148	
Female	0.970 ± 0.051		0.946 ± 0.145	
Side of contrast injection		0.928		0.317
Right	0.972 ± 0.050		0.942 ± 0.131	
Left	0.972 ± 0.046		0.974 ± 0.177	
Name of contrast agent		0.073		0.042^*^
Iomeprol	1.002 ± 0.037		0.956 ± 0.128	
Iopamidol	0.962 ± 0.056		0.910 ± 0.143	
Iohexol	0.974 ± 0.038		0.993 ± 0.154	
Ioversol	0.977 ± 0.038		1.023 ± 0.125	
Density of contrast agent		0.254		0.052
300 mgI/ml	0.955 ± 0.048		0.883 ± 0.078	
320 mgI/ml	0.977 ± 0.038		1.023 ± 0.125	
350 mgI/ml	0.981 ± 0.039		0.984 ± 0.141	
370 mgI/ml	0.962 ± 0.057		0.913 ± 0.148	

For age, Pearson’s correlation coefficients (with 95% confidence interval) are shown. For other factors, mean ± standard deviation values are described. Student’s *t* test was used for the comparison of TD_PAU_ and BLV_PAU_ between categories for sex and side of contrast injection. Analysis of variance was performed for the comparison of TD_PAU_ and BLV_PAU_ among categories for name of contrast agent and density of contrast agent. BLV_PAU_, ratio of CT pulmonary angiography to unenhanced for both lung volume; TD_PAU_, ratio of CT pulmonary angiography to unenhanced for tracheal diameter

^*^Statistically significant

## Discussion

This study revealed that iodine contrast agent injection reduced tracheal diameter and both lung volumes in CT pulmonary angiography compared with unenhanced CT. The decrease in both lung volumes was large for certain types of iodine contrast agents.

Our study revealed a decrease in tracheal diameter and both lung volumes at the timing of CT pulmonary angiography compared with unenhanced CT, both of which were scanned at end-inspiratory level. Our study would be in line with a previous article, which reported that the tracheal diameter and both lung volume at the arterial phase were transiently reduced [4]. The difference between the current study and that previous article lies in the scan timing. CT pulmonary angiography is usually aimed to scan at the timing when CT attenuation of the pulmonary artery is most likely to increase, which is earlier than CT angiography. This indicates that the decrease of both lung volume and tracheal diameter can be seen quite quickly after starting the contrast agent injection, or more precisely when the majority of contrast agents are on the right side of the heart.

Moreover, this study revealed that certain types of iodine contrast agents had a larger impact on CT pulmonary angiography (*p* = 0.042). This study used two different iodine contrast densities (300 and 370 mgI/ml) of iopamidol, and BLV_PAU_ values was relatively low for both (0.883 for 300 mgI/ml and 0.913 for 370 mgI/ml) compared with those for other contrast agents (0.956–1.023). Differences in the molecular structure of iodine contrast agents might be associated with the degree of decrease in both lung volumes at CT pulmonary angiography. However, the precise mechanism remains unclear.

Previous studies have revealed that the ratios of lower lobe volume at the expiratory level to those at the inspiratory level were smaller than the ratios of upper or middle lobes (41.1%–41.7% *vs.* 51.9%–65.4% [19] or 57.4%–57.8% *vs.* 67.5%–74.1% [15]). The present study revealed smaller ratios for the CT pulmonary angiography to unenhanced CT of the lower lobe volume (0.963 for the right and 0.921 for the left), which is located near the diaphragm, than those of the upper or middle lobe volume (0.968–1.006 for the right and 0.945 for the left). This indicates that the decrease in lung volume at CT pulmonary angiography was caused by the degree of inspiration level and not merely by increased CT attenuation of the vessels. This is supported by the two other data, a decrease in tracheal diameter observed at CT pulmonary angiography and a significant positive correlation observed between TD_PAU_ and BLV_PAU_.

This study has some limitations. First, CT pulmonary angiography and unenhanced CT were not scanned simultaneously. However, we made efforts to omit the apparent factors affecting lung volume by including patients without acute lung or pleural lesions and those with a time interval of less than 365 days between CT pulmonary angiography and unenhanced CT. The actual median time interval between CT pulmonary angiography and unenhanced CT was short with 50 days. Second, the slice thickness and interval of CT images were relatively large. However, this would not have affected the main results of our study because these parameters were the same between CT pulmonary angiography and unenhanced CT. Third, data were analyzed retrospectively. Even under an instruction of deep inspiration, the inspiration level can vary with patient’s symptoms or comorbidities. Therefore, a further study with prospective CT acquisition would be necessary to validation the phenomenon observed in our study. Finally, our study was not focused on including patients with diseases, such as interstitial pneumonia, for which lung volumetry plays important roles; thus, the impact of this phenomenon on the evaluations of such diseases was not assessed. Future investigation regarding this topic would be necessary.

In conclusion, iodine contrast agent rapid infusion significantly reduced tracheal diameter and both lung volumes in CT pulmonary angiography compared with unenhanced CT. Certain types of iodine contrast agents have a relatively large effect on both lung volumes.
